# Suicide Attempt With Levothyroxine Overdose

**DOI:** 10.7759/cureus.36172

**Published:** 2023-03-15

**Authors:** Angad S Gill, Harpreet K Rai, Abhijana Karunakaran, Ajay Chaudhuri

**Affiliations:** 1 Internal Medicine, Griffin Hospital, Derby, USA; 2 Division of Endocrinology and Metabolism, Jacobs School of Medicine, University at Buffalo, Buffalo, USA

**Keywords:** drug overdose, thyroid hormone overdose, poisoning, levothyroxine, levothyroxine poisoning, levothyroxine overdose

## Abstract

Symptoms of levothyroxine overdose may vary depending on age, metabolism, etc. There are no specific guidelines for treating levothyroxine poisoning. Here, we present the case of a 69-year-old man with a history of panhypopituitarism, hypertension, and end-stage renal disease who attempted suicide by ingesting 60 tablets of 150 µg levothyroxine (9 mg). Upon presentation to the emergency room, he was asymptomatic despite the free thyroxine level above the range of the assay. During the hospital stay, he developed sinus tachycardia, which was controlled with propranolol. Mild elevations in liver enzymes were also noted. He received stress-dose steroids; hemodialysis was performed a day earlier, and cholestyramine was administered. Thyroid hormone levels started to improve by day seven and finally normalized in 20 days, after which the home dose of levothyroxine was resumed. The human body has several mechanisms to compensate for levothyroxine toxicity, including the conversion of excess levothyroxine to inactive reverse triiodothyronine, increased binding to thyroid-binding globulin, and hepatic metabolism. This case shows that it is possible to have no symptoms even with an overdose of up to 9 mg a day of levothyroxine. Signs and symptoms of levothyroxine toxicity may not appear for several days after ingestion, and, therefore, close observation preferably on a telemetry floor is recommended until the thyroid hormone levels start to decrease. Effective treatment options include beta-blockers preferably propranolol, early gastric lavage, cholestyramine, and glucocorticoids. While hemodialysis has a limited role, antithyroid drugs and activated charcoal are ineffective.

## Introduction

About four in 100 people in the United States have hypothyroidism [1]. Levothyroxine is the first-line drug for the treatment of hypothyroidism [2]. Massive amounts of levothyroxine may be ingested intentionally or accidentally. The most common intentional reasons include weight loss and suicidal ideation among others. A potentially toxic dose includes acute ingestion of more than 5 mg of levothyroxine or 0.75 mg of triiodothyronine [3]. Given the varied presentations of levothyroxine overdose and a lack of clear benefits of various treatment modalities, treatment of levothyroxine poisoning is challenging, and there are no specific guidelines available. Here, we present a case of levothyroxine overdose in a 69-year-old male with ingestion of 9 mg of levothyroxine.

## Case presentation

A 69-year-old male with a past medical history of panhypopituitarism due to pituitary resection for a non-secreting pituitary adenoma, hypertension, end-stage renal disease, and depression presented with ingestion of 60 tablets of 150 µg levothyroxine (9 mg) as a suicide attempt. His home medications included levothyroxine 150 µg daily, hydrocortisone 20 mg in the morning and 10 mg in the afternoon, testosterone 200 mg intramuscular injection every two weeks, bupropion 150 mg daily, and enalapril 10 mg daily.

The patient presented to the emergency room (ER) four hours after ingestion of levothyroxine pills and was asymptomatic on presentation. The physical examination was negative for any tremors, lid lag, and hyperactive deep tendon reflexes. Vital signs included a blood pressure of 154/90 mmHg and a heart rate of 85 beats/minute. He was afebrile and oxygen saturation was good on room air. An electrocardiogram revealed sinus rhythm. The patient’s laboratory workup was significant for free thyroxine (FT4) of >7.04 ng/dL (normal range: 0.93-1.70 ng/dL), total thyroxine (TT4) of >24.9 ng/dL (normal range: 4.6-12.0 ng/dL), total triiodothyronine (TT3) of 171 ng/dL (normal range: 80-200 ng/dL), and thyroid-stimulating hormone (TSH) of <0.02 mU/L (normal range: 0.27-4.20 mU/L). Given known hypopituitarism, evaluation and treatment decisions were made based on thyroxine and triiodothyronine levels rather than TSH.

The patient received activated charcoal in the ER and was closely monitored with telemetry for the development of any arrhythmia. Upon admission, 100 mg of intravenous (IV) hydrocortisone was administered as a stress dose of steroids which was titrated down to his home dose. Hemodialysis was performed a day earlier than his regular schedule. The following day he developed sinus tachycardia with a heart rate of 110 beats/minute and was started on propranolol 10 mg three times a day. During the hospital course, thyroid function tests (TFT) (Table 1) were closely monitored. Mild elevation in liver enzymes was noted which peaked on day five with aspartate transaminase of 70 U/L (normal range: 5-40 U/L) and alanine transaminase of 69 U/L (normal range: 5-50 units/L). Cholestyramine was administered on day three of the hospitalization, but given the worsening of liver enzymes, it was soon discontinued. TT3 reached a peak value of 291 ng/dL on day three and eventually normalized within a week of the overdose. FT4 remained high (>7.04 ng/dL) for the first week and slowly normalized to within normal limits (1.36 ng/dL) on day 19. The patient was noted to have developed sinus tachycardia on day two with his heart rate going up to 130s by day six. The propranolol dose was accordingly titrated to 40 mg three times a day. Heart rate improved to 80s by day seven, coinciding with decreasing FT4 and TT3 (Table 1), and propranolol was tapered off. Eventually, the home dose of levothyroxine was resumed on day 20.

**Table 1:** Thyroid hormone levels, liver enzyme levels, and heart rate measurements from day one to day nine. TSH: thyroid-stimulating hormone; T4: thyroxine; T3: triiodothyronine; AST: aspartate transaminase; ALT: alanine transaminase; NA: value not available

Parameter (reference range)	Day 1	Day 2	Day 3	Day 4	Day 5	Day 6	Day 7	Day 8	Day 9
TSH (0.27–4.20 mU/L)	<0.02	<0.02	<0.02	<0.02	<0.02	<0.02	<0.02	<0.02	<0.02
Free T4 (0.93–1.70 ng/dL)	>7.04	>7.04	>7.04	>7.04	>7.04	>7.04	6.26	4.32	3.54
Total T3 (80–200 ng/dL)	171	224	272	291	272	NA	NA	181	151
Maximum heart rate (beats per minute)	89	110	100	120	110	130	90	90	80
AST (5–40 units/L)	25	NA	NA	NA	70	54	49	34	33
ALT (5–50 units/L)	21	NA	NA	NA	69	57	58	48	48

## Discussion

Here, we present a case of massive levothyroxine overdose with a relatively benign clinical course. Levothyroxine is not entirely dissolved by the gastric juices and only 10-15% is absorbed in the duodenum. It is mainly absorbed (>53%) in the jejuno-ileum, thus leading to a progressive rise in both TT3 and total T4 levels in the first 24 hours after the ingestion. Moreover, FT4 needs to be converted to the active free T3, thus delaying the onset of symptoms for several days after the ingestion. Peak thyroxine plasma concentration can occur even two to four days after ingestion [4]. Our body has powerful and interesting pathways to compensate for levothyroxine toxicity to the point of showing no remarkable symptoms. First, the excess levothyroxine can be converted to biologically inactive reverse triiodothyronine, which, in turn, can inhibit levothyroxine toxicity by competitively binding to the receptor [5]. Second, levothyroxine can bind to certain proteins such as thyroid-binding globulin, preventing it from becoming biologically active [6]. Third, the liver may metabolize excessive levothyroxine, thus reducing its effects [7].

Given a long 7.5-day half-life of levothyroxine [8], all cases with levothyroxine toxicity should be monitored closely, preferably with telemetry given the possibility of developing tachyarrhythmias or seizures. Beta-blockers are a preferred treatment strategy given their additional role in inhibiting the peripheral conversion of thyroid hormones [9]. Additionally, propranolol has also been shown to decrease serum T3 concentration, especially at high doses (>160 mg/day) [10]. Sinus tachycardia can be treated with oral propranolol 0.1-0.5 mg/kg every four to six hours. Tachyarrhythmias can either be treated with IV propranolol 0.01-0.1 mg/kg repeated every two to five minutes to the desired effect or with IV esmolol 0.025-0.1 mg/kg/minute. Activated charcoal administration, although a common management practice, has been ineffective for levothyroxine overdose in reported cases even with repeated administrations [11]. Gastric lavage may help in cases with early presentation of massive levothyroxine toxicity (e.g., >10,000 µg) [6]. Another option is cholestyramine, an ion exchange resin that binds thyroxine to help with its elimination [12]. It is important to monitor liver enzymes as cholestyramine is hepatotoxic. Glucocorticoids (dexamethasone 4 mg orally) decrease the conversion of T4 to active hormone T3 [3] and are recommended especially in patients with a massive levothyroxine overdose (e.g., >10,000 µg) or if the free T4 is higher than the limit of quantification of the lab. Antithyroid drugs are ineffective in cases of overdose as endogenous thyroid hormone production is already suppressed. However, propylthiouracil may be used to block T4 to T3 conversion, although its role at such a high load of levothyroxine may be limited [12]. Our patient also received hemodialysis a day earlier, but the role of hemodialysis in levothyroxine overdose is limited given that most of the levothyroxine is bound to protein and not dialysable [12]. Iopanoic acid and sodium ipodate reduce peripheral conversion of T4 to T3 but the data on their use is limited [4]. There are other possible symptoms and signs reported in other studies, including seizures, successfully treated with phenytoin and phenobarbital, or hyperthermia treated with acetaminophen [13,14]. Plasmapheresis has shown mixed results [15-17], but given that our patient was asymptomatic upon presentation, this was not pursued. Experimental data on cathartics in combination with activated charcoal has shown conflicting results and is not endorsed by the American Academy of Clinical Toxicology [18]. Fortunately, our patient did not develop any other complications.

Given the rarity of levothyroxine toxicity, this case would help physicians in learning more about the possible presentation and management options of levothyroxine toxicity and help develop management guidelines for levothyroxine toxicity in the future.

## Conclusions

Patients may remain asymptomatic even with a levothyroxine overdose of up to 9 mg in a day. As no standard guidelines are currently available, the management of levothyroxine overdose should be individualized based on the amount ingested, as well as the clinical symptoms and signs of the patient. Patients should be monitored for cardiac arrhythmias and ideally kept under observation on a telemetry floor until their thyroid hormone levels begin to decrease. Beta-blockers (preferably propranolol) are the preferred treatment modality. Early gastric lavage, glucocorticoids, and cholestyramine are also effective options. Activated charcoal and hemodialysis have no role in levothyroxine overdose.
